# The Effects of Adding Extruded Highland Barley Flour on the Thermomechanical Properties of Wheat Flour Dough and the Overall Quality of Fresh Wet Noodles

**DOI:** 10.3390/foods13193105

**Published:** 2024-09-28

**Authors:** Yiqing Zhu, Xuecong Wang, Xinyu Zhang, Yan Du, Feng Liang, Fan Zhang, Chongyi Wu, Qingyu Zhao, Qun Shen

**Affiliations:** 1 College of Food Science and Nutritional Engineering, China Agricultural University, National Center of Technology Innovation (Deep Processing of Highland Barley) in Food Industry, National Engineering Research Center for Fruit and Vegetable Processing, Beijing 100083, China; yiqingzhu@163.com (Y.Z.); wangxuecong17@163.com (X.W.); xinyuzhang0319@163.com (X.Z.); zhangfan3021@163.com (F.Z.); 13616617052@163.com (C.W.); zqy565527877@163.com (Q.Z.); 2 Qinghai Tianyoude Technology Investment Management Group Co., Ltd., Qinghai Engineering Technology Research Institute for Comprehensive Utilization of Highland Barley Resources, Xining 810016, China; duyan_tyd@163.com (Y.D.); liangfeng@qkj.com.cn (F.L.); 3 Beijing Industrial Technology Research Institute Ltd., Beijing 101111, China

**Keywords:** extruded highland barley flour, thermomechanical properties, quality characteristics, fresh wet noodles, starch digestion

## Abstract

This study examined how adding extruded highland barley flour (EHBF) affects the thermomechanical properties of wheat flour dough and the overall quality of fresh wet noodles. EHBF increased the gel strength and pasting temperature of wheat flour compared to regular highland barley flour. Moreover, higher EHBF levels reduced dough development time and stability time. EHBF improved the color and springiness of fresh wet noodles and decreased their cooking time and light transmittance relative to the color and springiness of the noodles in the control group. Notably, noodles with 20% EHBF showed a compact microstructure and received the highest sensory evaluation score. Adding EHBF lowered the estimated glycemic index of fresh wet noodles by reducing rapidly digestible starch and increasing slowly digestible starch and resistant starch contents. Thus, EHBF is a promising functional ingredient for enhancing the quality of fresh wet noodles.

## 1. Introduction

As a traditional staple food in many Asian countries, noodles boast a history spanning over 4000 years [1]. Noodles are composed of wheat flour, water, and salt, and can be categorized into dry, semi-dry, and fresh wet types based on their moisture content [2]. Fresh wet noodles offer convenient processing, excellent taste, and cost-effectiveness [3,4]. However, as consumer demand shifts towards healthier options, traditional fresh wet noodles may not meet health expectations [5,6]. Hence, new functional varieties of fresh wet noodles need to be developed using wheat flour as a base.

Highland barley (*Hordeum vulgare* L.), known as Qingke, is an important crop native to the Qinghai-Tibet Plateau in China [7]. Recently, its functional components, such as γ-amino butyric acid, dietary fiber (especially β-glucan), and phenolic compounds, have gained attention [8,9]. Several studies have investigated ways to improve noodle quality by incorporating highland barley flour into the dough. For example, Montalbano et al. [10] found that ditalini pasta made from a blend of durum wheat and barley flour showed significantly higher total phenol content and antioxidant capacity compared to the control (*p* < 0.001). Zhao et al. [11] demonstrated that adding flour from hull-less barley produced high-quality noodles rich in dietary fiber without the need for chemical additives. Along with its nutritional benefits, highland barley is linked to various health benefits, including the prevention of cardiovascular disease, diabetes, and obesity, as suggested by epidemiological studies [12,13].

Despite the health benefits of highland barley flour as a promising ingredient for noodle production, less gluten protein makes it difficult to form a strong network structure needed to maintain noodle quality [14]. Heat treatment, such as starch gelatinization through methods like extrusion, is effective in enhancing dough network formation [1]. Common heat treatment techniques include extrusion, steaming, baking, pasting, and microwaving [8], with extrusion being favored in grain processing due to its cost-effectiveness and efficiency [15]. Extrusion involves the gelatinization of starch through mechanical shear, temperature, humidity, and pressure, which can improve its physical and chemical properties [16]. Studies have demonstrated that incorporating extruded flours like buckwheat [17], rice [18], mung bean [19], and black soybean [20] can enhance dough properties and noodle quality. Some studies have highlighted the preparation of nutrient-rich fresh wet noodles by mixing extruded highland barley flour with raw highland barley flour and wheat flour at a 1:1:3 ratio [4,5]. However, most studies on fresh highland barley noodles predominantly investigated shelf life, with limited research on potential health benefits such as in vitro starch digestion and estimated glycemic index. Moreover, comprehensive comparisons between extruded and unextruded highland barley flour regarding their effects on dough properties and fresh wet noodle quality remain scarce.

Thus, in this study, we aimed to achieve two main objectives: (I) evaluate the feasibility of preparing fresh wet noodles using varying amounts of extruded highland barley flour (0%, 10%, 20%, 30%, and 40%) and (II) compare the effects of extruded versus unextruded highland barley flour on the mixing behavior, microstructure, color changes, cooking quality, texture, sensory attributes, and in vitro starch digestibility of fresh wet noodles. These results are intended to act as a reference for the application of highland barley in the food industry.

## 2. Materials and Methods

### 2.1. Materials

Highland barley (*Hordeum vulgare* L. Zangqing 320) was provided by the Tibet Academy of Agriculture and Animal Husbandry Sciences (Nyingchi, China). High-gluten wheat flour was purchased from WUDELI Flour Mill Group Corp. (Handan, China). Porcine pancreatic α-amylase (S31302, ≥5 u/mg solid) and amyloglucosidase (S10017, ≥100 units/mg) were obtained from Shanghai Yuanye Biotechnology Co., LTD (Shanghai, China). Other chemical reagents were analytical grade and purchased from Solarbio Science &Technology Co., Ltd. (Beijing, China).

### 2.2. Preparation and Chemical Analysis of Flour

Highland barley flour (HBF) was obtained through roller milling and sieved through an 80 mesh screen. For extruded highland barley flour (EHBF), the highland barley was initially pulverized into flour before undergoing twin-screw extrusion treatment [8]. EHBF was subsequently produced by roller milling and sieved through an 80 mesh screen.

The proximate composition of flour samples was determined according to AACC approved methods [21]. HBF contained 12.04% moisture, 9.03% protein, 1.82% fat, 82.78% starch, 28.42% damaged starch, 39.66% amylose, 1.14% crude fiber, and 1.18% ash. EHBF contained 9.19% moisture, 11.46% protein, 1.35% fat, 75.64% starch, 38.93% damaged starch, 35.68% amylose, 2.53% crude fiber, and 1.91% ash. High-gluten wheat flour contained 13.59% moisture, 13.29% protein, 0.89% fat, 81.71% starch, 27.37% damaged starch, 59.26% amylose, 0.46% crude fiber, and 0.37% ash.

### 2.3. Pasting Properties

The pasting properties of the flour sample were assessed using a Rapid Visco Analyser (RVA, Model Super3, Newport Scientific, Warriewood, Australia). Each 3 g of sample (based on 14% moisture content) was mixed with 25 mL of deionized water in an aluminum container. The mixture was allowed to stabilize at 50 °C for 1 min, heated to 95 °C at a rate of 12 °C/min, held at 95 °C for 2.5 min, cooled back to 50 °C at the same rate, and maintained at 50 °C for 2 min. Key pasting parameters were recorded: peak viscosity, trough viscosity, breakdown value, final viscosity, setback value, and pasting temperature [22].

### 2.4. Mixolab Measurements

The mixing behaviors of the dough sample were assessed using the Mixolab (Chopin, Tripette et Renaud, Paris, France). Each sample of flour and deionized water (total weight: 75 g) was placed in a Mixolab bowl. Testing began when the maximum torque C1 reached 1.10 ± 0.05 Nm. Parameters such as water absorption (%), protein weakening (C2, Nm), starch gelatinization (C3, Nm), starch retrogradation (C5, Nm), dough development time (min), and dough stability time (min) were derived from the recorded curve [23].

### 2.5. Preparation of Fresh Wet Noodles

Fresh wet noodles were prepared following previous methods with some modifications [3]. Initially, a mixture of 100 g of flour and 45 mL of deionized water was stirred for 6 min using a small-scale kneader (Hauswirt, Hong Kong, China). The resulting dough was incubated in an electric thermostatic incubator (Boxun, Shanghai, China) at 30 °C for 30 min. Noodle strands (10.0 cm in length, 4.0 mm in width, and 1.5 mm in thickness) were extruded using a laboratory noodle machine (Model JMTD-168/140, Beijing, China).

### 2.6. Scanning Electron Microscopy (SEM)

Micrographs of the noodle samples were obtained via SEM (Hitachi Ltd., Tokyo, Japan). The freeze-dried noodle samples were gently broken by hand. The noodle sample cross-sections were coated with a thin layer of gold via an ion sputter coater. A representative image was captured at an operating voltage of 5.0 kV and a magnification of 500× [17].

### 2.7. Color Measurements

The color change in the noodle sample was measured using a chroma meter (Konica Minolta CR-400, Osaka, Japan). The color parameters L* (lightness/darkness), a* (redness/greenness), b* (yellowness/blueness), and ΔE (total color difference) were used to characterize the color of noodle samples [24]. The noodle sample was placed on a horizontal operating table, and the chroma meter was calibrated using a standard whiteboard (L* = 94.40; a* = 0.45; b* = 0.75). Five random measurement points were selected for each sample against the standard whiteboard background.

### 2.8. Cooking Characteristics

The cooking quality of the noodle samples was assessed according to the method described by Fu et al. [1] with modifications. Fresh noodle samples (10 g each) were weighed and boiled in 500 mL water. The optimal cooking time was recorded when the white core of the noodle disappeared. Another set of 10 g of noodle samples was cooked in 500 mL of boiling water at this optimal time. The percentage of broken noodles relative to the initial dry weight was calculated by counting the broken noodles. The transmittance of the cooking water was measured at 620 nm using a UV–visible spectrophotometer (Hitachi High-Technologies, Tokyo, Japan).

### 2.9. In Vitro Starch Digestibility

The in vitro starch digestibility of the noodle samples was evaluated based on the method described by Yu et al. [25]., with minor adjustments. Initially, 0.2 g of cooked noodles was dispersed in 20 mL of sodium acetate buffer (0.2 mol/L, pH 5.2) containing porcine pancreatic α-amylase (170 U/mL) and amyloglucosidase (10,000 U/mL). The enzymatic digestion was performed at 37 °C in a shaking water bath at 200 rpm. At specific time intervals (0, 20, 60, 90, 120, and 180 min), 0.2 mL of the digestion solution was withdrawn and transferred to a centrifuge tube containing 1 mL of ethanol. After centrifugation at 1500× *g* for 10 min, the glucose content in the supernatant was quantified using a D-glucose assay kit (GOPOD, Megazyme, Ireland). The experimental data were analyzed using nonlinear models to determine starch hydrolysis kinetics parameters such as rapidly digested starch (RDS), slowly digested starch (SDS) and resistant starch (RS) [6]. The estimated glycemic index (*e*GI) is calculated as follows:

*e*GI = (0.549 × HI) + 39.71

where the hydrolysis index (HI) was obtained by dividing the area under the hydrolysis curve of each sample by the corresponding area of white bread (defined as 100%).

### 2.10. Textural Analysis

The textural characteristics of the noodle samples were evaluated using a TA texture analyzer, XT Plus (Stable Micro Systems Ltd., Godalming, UK). Cooked noodle samples were evenly cut into 2 cm segments and compressed to 70% using a P36/R probe at a force of 5 g. The compression test parameters included a test speed of 2.0 mm/s and a residence time of 5 s. Each test was repeated five times, and relevant parameters were extracted from the force-time curve [26].

### 2.11. Sensory Evaluation

The sensory evaluation of the noodle samples was conducted at the College of Food Science and Nutritional Engineering, China Agricultural University. The evaluation involved 20 trained panelists (1:1 male-to-female ratio), each evaluated individually in booths. Each panelist received 10 g of cooked noodles, a glass of water, and a score sheet at the start of the evaluation process [20]. The total sensory evaluation score was 100, and the sensory attributes assessed included appearance (1–10), taste (1–20), color (1–10), firmness (1–25), smoothness (1–5), stickiness (1–25), and flavor (1–5).

### 2.12. Statistical Analysis

All statistical analyses were conducted using SPSS (version 25.0, IBM, New York, NY, USA). The data are presented as the mean ± standard deviation. Each experiment was replicated at least three times, and statistical significance was determined by performing a one-way analysis of variance (ANOVA) with Tukey’s post-hoc test (*p* < 0.05).

## 3. Results and Discussion

### 3.1. Effects of HBF and EHBF Addition on the Pasting Properties of Wheat Flour

The RVA pasting characteristics of wheat flour mixed with different contents of highland barley flour (HBF) and extruded highland barley flour (EHBF) are detailed in Table 1. Increasing the amounts of HBF and EHBF led to a decrease in the peak viscosity of wheat flour. Peak viscosity reflects the rupture of starch granules and is related to the leaching of amylose molecules in a system [7]. The content of amylose in HBF (39.66%) and EHBF (35.68%) was lower than that of wheat flour (59.26%), resulting in a decrease in the peak viscosity of wheat flour (Section 2.2). In addition, lipids may also affect peak viscosity. Fu et al. [1] reported that lipids can combine with amylose to form complexes, limiting amylose leaching and thus reducing the peak viscosity of buckwheat flour. Similarly, the trough viscosity, final viscosity, and setback values also decreased. A decrease in amylose content weakened the swelling capacity of wheat flour, resulting in lower trough viscosity, final viscosity, and setback value [27]. Breakdown value is a measure of the degree of disintegration or stability of starch granules. The addition of HBF promoted the breakdown value of wheat flour, indicating that HBF had poor resistance to breakdown during heating and shearing [19].

Compared to HBF, EHBF significantly lowered the breakdown value of wheat flour at the same addition level (*p* < 0.05). This effect may be attributed to the gelatinization of highland barley starch during extrusion. Pre-gelatinization reduced the swelling power of highland barley starch, thus decreasing the breakdown value of wheat flour [16]. The pasting temperature reflects the temperature at which the viscosity of wheat flour begins to increase during heating [22]. This increase suggested that wheat flour containing EHBF is more resistant to gelatinization than that containing HBF at the same addition level. It is not difficult to understand that the pre-gelatinized highland barley starch in EHBF would limit the gelatinization of wheat starch during heating, which is consistent with the results of previous studies [24]. A study found that moderate extrusion can strengthen the rigidity of starch granules and improve the interaction within the starch chain in the amorphous region [16]. Consequently, the peak viscosity, trough viscosity, and final viscosity of the wheat flour containing EHBF were found to be greater than those containing HBF. These results matched the observed changes in pasting temperature.

### 3.2. Effects of HBF and EHBF Addition on the Mixolab Parameters of Wheat Flour Dough

Mixolab measurements of wheat flour dough mixed with different contents of HBF and EHBF are shown in Table 2. The water absorption of wheat flour dough decreased after HBF was added, compared to that observed in the control group. This outcome may be attributed to alterations in gluten formation, which is the primary factor influencing dough water absorption [28]. The water absorption of the wheat flour dough started increasing with an increase in EHBF. A study found that starch granules gelatinize during extrusion, leading to higher water adsorption [29]. Simultaneously, the extrusion treatment exposed the hydroxyl groups in HBF, facilitating increased interaction of water molecules through hydrogen bonding [15].

The C2 value was related to protein content and protein denaturation, which indicated protein weakening [23]. Compared to the control group, incorporating HBF and EHBF decreased the C2 value of wheat flour dough. This effect may arise from gluten protein dilution, weakening protein [28]. As the addition of HBF and EHBF increased, the C2 value of wheat flour dough did not change significantly. This phenomenon may be due to the fact that HBF and EHBF contain more dietary fiber, which is beneficial in delaying protein denaturation during heating [30]. Compared with HBF, EHBF addition significantly decreased the C2 value of wheat flour dough. Extrusion caused protein denaturation in EHBF, leading to protein weakening [31].

The C3 and C5 values are indicators of starch gelatinization and retrogradation [20]. Adding HBF did not significantly change the C3 value of the wheat flour dough compared to that recorded in the control group, whereas adding EHBF decreased the C3 value, suggesting that HBF had stronger swelling power during heating and gelatinization [26]. It is not difficult to understand that extrusion can pre-gelatinize EHBF, inhibit the reswelling of starch granules, and reduce the degree of starch gelatinization [24]. After cooling the temperature to 50 °C, including HBF and EHBF decreased the C5 value of wheat flour dough. This decrease suggested a lower degree of starch retrogradation in the dough [32]. However, EHBF significantly decreased the C5 value compared to HBF (*p* < 0.05), probably due to its high water absorption capacity, which helped retain moisture in the dough and delayed retrogradation [31].

Regarding the development time of dough, the incorporation of HBF and EHBF shortened the time required to achieve maximum consistency in wheat flour dough. Typically, dough development time is associated with the gluten formation present in the system [32]. Adding HBF and EHBF decreased the relative protein content and gluten dilution. EHBF notably decreased dough development time at 10–30% addition level compared to HBF (*p* < 0.05), probably due to enhanced water absorption of the starch by extrusion treatment, which shortened dough development [29]. However, at a 40% addition level, HBF also shortened dough development time, probably due to physical interference causing dough weakening [31]. Dough stability with HBF resembled water absorption characteristics. The decrease in dough stability may stem from gluten dilution, while any increase could be attributed to dietary fiber. Han et al. [30] reported that a certain percentage of dietary fiber can interact with gluten protein to maintain the stability of Tartary buckwheat dough. In addition, EHBF addition significantly decreased dough stability compared to HBF (*p* < 0.05). It is not difficult to understand that the extrusion destroys the integrity of the starch granules, promotes the water absorption of the dough, and affects the stability of the dough.

### 3.3. Effects of HBF and EHBF Addition on the Microstructure of Fresh Wet Noodles

**Figure 1:**
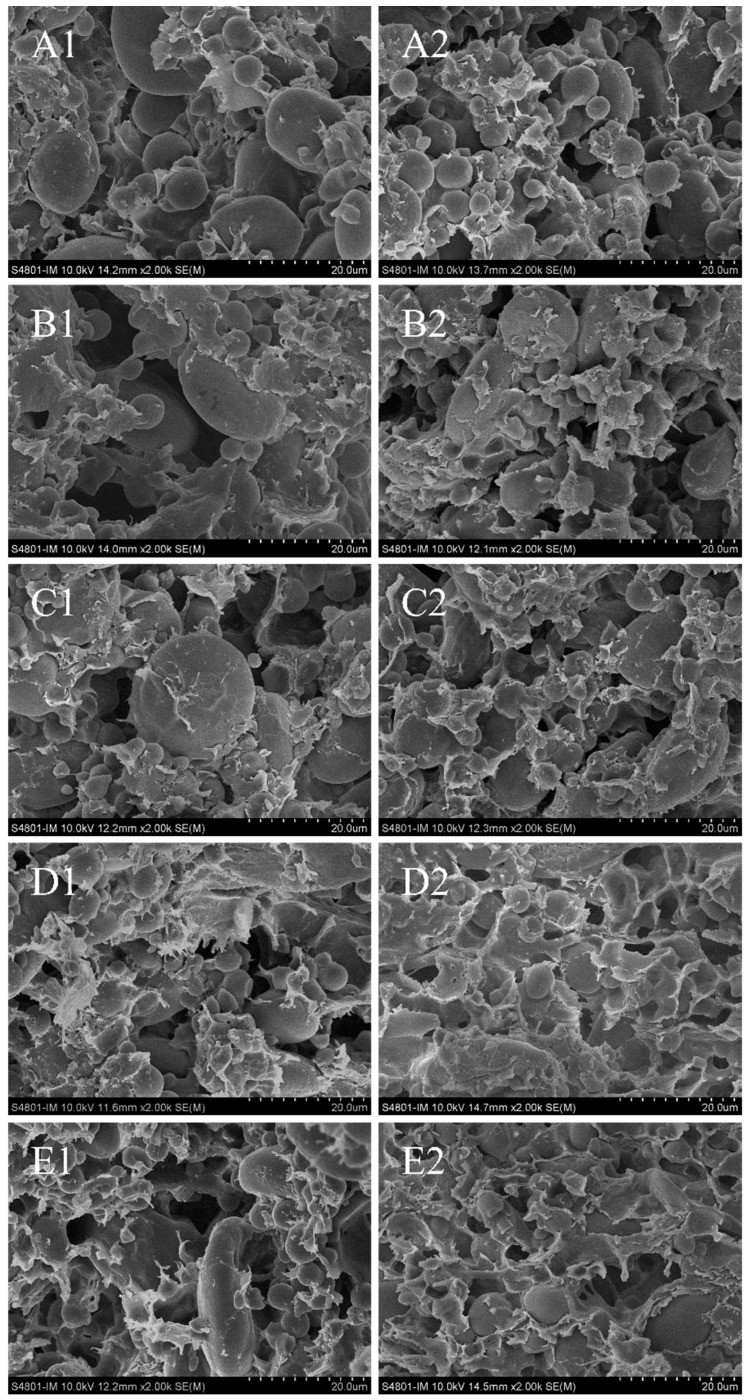
Effects of HBF and EHBF addition on the microstructure of fresh wet noodles. (**A1**–**E1**) represent the fresh wet noodles with 0, 10, 20, 30, and 40% addition of HBF, respectively. (**A2**–**E2**) represent the fresh wet noodles with 0, 10, 20, 30, and 40% addition of EHBF, respectively. HBF: highland barley flour; EHBF: extruded highland barley flour.

The microstructure of fresh wet noodles with varying additions of HBF and EHBF was analyzed using SEM. The microstructure of the control group was dense, with starch granules enveloped by the network structure of gluten protein (Figure 1). As the levels of HBF and EHBF increased, the continuity of the gluten network decreased, and the porosity of the noodle matrix increased. The structure of the fresh wet noodles appeared less compact, probably due to the diluting influence of HBF and EHBF on gluten. A study found that the incorporation of hull-less barley flour decreased the uniformity of gluten protein, resulting in a higher number of starch granules separated from the gluten network [11]. When less than 20% HBF and EHBF were incorporated, the microstructure of the fresh wet noodles resembled that of the control group. This observation suggested that a suitable level of HBF and EHBF inclusion can preserve the continuity of the gluten network and the encapsulation of starch granules within the noodle matrix [25]. When the level of HBF or EHBF exceeded 20%, the noodle matrix became loose and discontinuous. EHBF, compared to HBF, led to reduced porosity in the fresh wet noodles. This alteration in microstructure may be associated with the breakdown of a starch structure during extrusion. Ma et al. [31] reported that black rice starch lost its original structure and converted into a paste during extrusion, leading to fewer pores in the wheat flour dough. Additionally, gelatinized starch acts as a hydrocolloid within the noodle matrix, aiding in enhancing the gluten network’s continuity [17]. Consequently, fresh wet noodles with added EHBF exhibited a more compact microstructure and improved water retention capability.

### 3.4. Effects of HBF and EHBF Addition on the Color Characteristics of Fresh Wet Noodles

**Figure 2:**
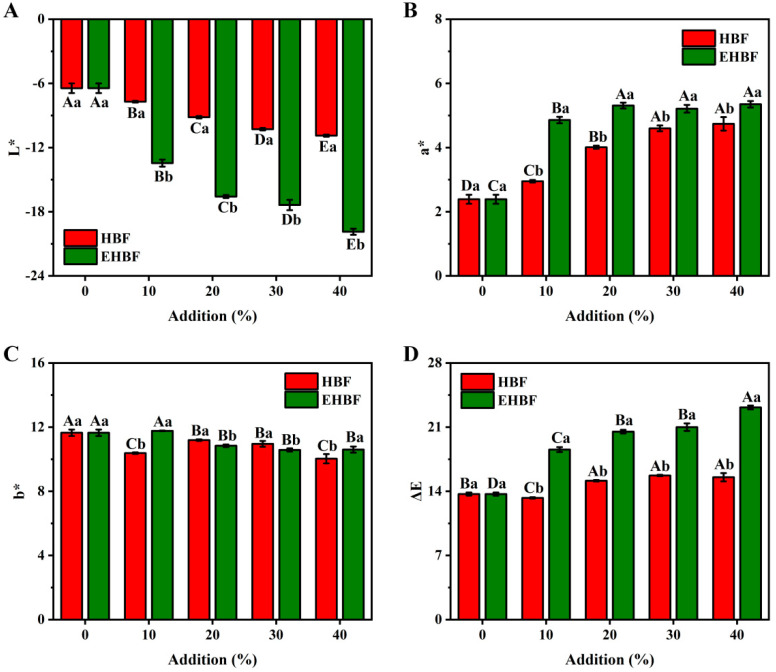
Effects of HBF and EHBF addition on the color parameters of fresh wet noodles. Values show mean ± standard deviation (n = 3). Different samples with the same addition level are marked with different small letters (a, b) for the expression of the significant difference (*p* < 0.05). The same sample at different addition level is marked with different capital letters (A, B, C, D, and E) for the expression of the significant difference (*p* < 0.05). HBF: highland barley flour; EHBF: extruded highland barley flour.

Color significantly influences consumer acceptance of fresh wet noodles [3]. The inclusion of HBF and EHBF in the noodle formulation led to a reduction in the L* value of fresh wet noodles, indicating a decrease in lightness (Figure 2A). The L* value, representing the lightness, is inversely related to the ash content in the flour. HBF and EHBF had a higher ash content than wheat flour, leading to the darkness of the fresh wet noodles [24]. Additionally, the incorporation of HBF and EHBF increased the a* value (redness) of the noodles, whereas the b* value (yellowness) showed minimal variation (Figure 2B,C). This change can be attributed to the phenolic compounds found in highland barley [9]. A study found that phenolic compounds significantly influence noodle color [20]. Compared to HBF, the addition of EHBF not only further decreased the L* value but also enhanced the a* value and the ΔE value of the noodles, signifying a darker and more reddish appearance (*p* < 0.05) (Figure 2D). This suggested that the extrusion process may enhance enzymatic or non-enzymatic browning reactions, leading to the formation of colored polymers [31]. Therefore, the amount of EHBF used to produce fresh wet noodles should be limited.

### 3.5. Effects of HBF and EHBF Addition on the Cooking Quality of Fresh Wet Noodles

**Figure 3:**
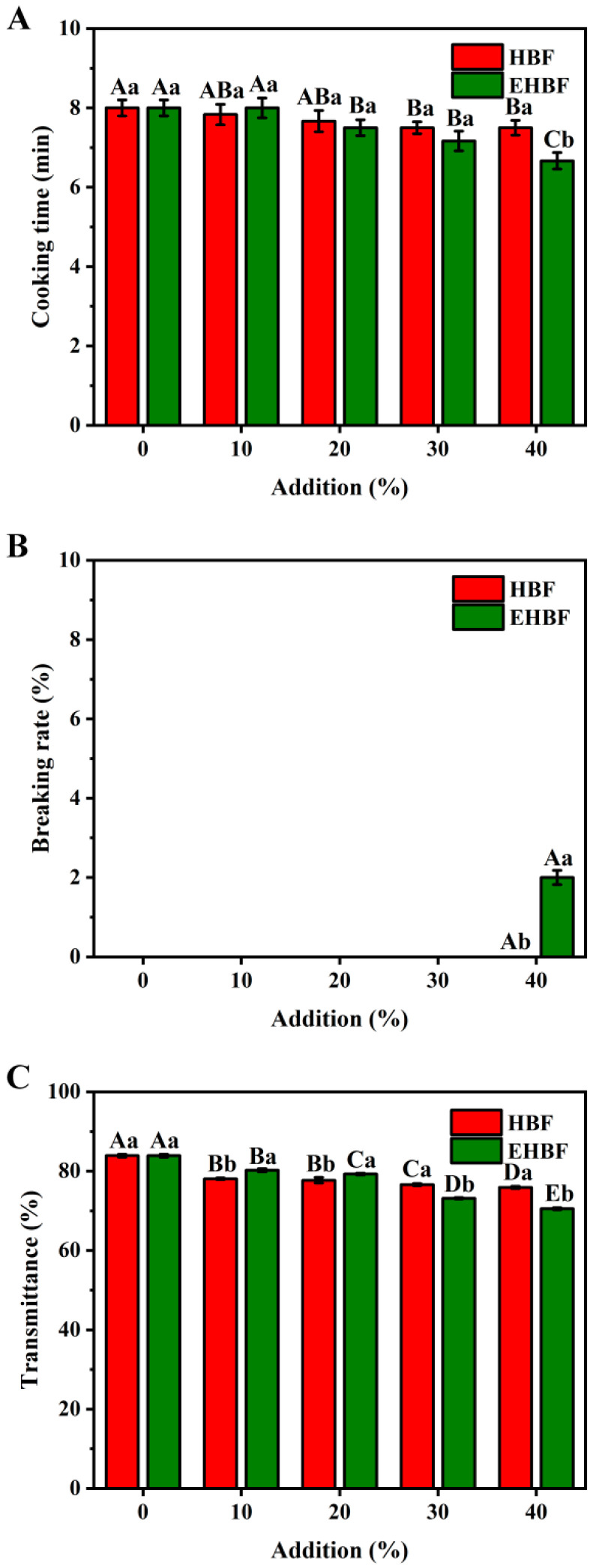
Effects of HBF and EHBF addition on the cooking quality of fresh wet noodles. Values show mean ± standard deviation (n = 3). Different samples with the same addition level are marked with different small letters (a, b) for the expression of the significant difference (*p* < 0.05). The same sample at different addition level is marked with different capital letters (A, B, C, D, and E) for the expression of the significant difference (*p* < 0.05). HBF: highland barley flour; EHBF: extruded highland barley flour.

The cooking quality of fresh wet noodles with different quantities of HBF and EHBF is presented in Figure 3. Compared to the control group, the inclusion of 30–40% HBF and EHBF reduced the optimal cooking time for fresh wet noodles (Figure 3A). The optimal cooking time is associated with the water absorption capacity of the dough [30]. Adding 30–40% HBF and EHBF, which indicates higher dietary fiber content, probably increased the water absorption of the dough through hydrogen bonding, consequently decreasing the cooking time required for the fresh wet noodles. 

The percentage of broken noodles is a crucial metric for assessing noodle quality [17]. After adding HBF and EHBF, the incidence of broken fresh wet noodles was 0% (Figure 3B). However, a 2% breakage rate was observed when 40% EHBF was added, suggesting that high levels of EHBF might compromise the stability of the dough, causing the fresh wet noodles to break more easily during cooking [1]. The transparency of the cooking water is an indicator of cooking loss in fresh wet noodles. A study found that the transparency of cooking water is inversely related to the cooking loss of noodles [24]. With the inclusion of HBF and EHBF, the transparency of cooking water decreased (Figure 3C). The addition of these flours may disrupt the protein network structure, facilitating the leaching of soluble substances from fresh wet noodles during cooking [33]. When 10–20% EHBF was added, the transparency of the cooking water was higher compared to that with HBF. This finding suggested that a lower addition of EHBF may stabilize the dough system more effectively, thus reducing the cooking loss of fresh wet noodles.

### 3.6. Effects of Adding HBF and EHBF on the Starch Digestibility of Fresh Wet Noodles

In vitro gastrointestinal digestion is used to simulate starch digestion in vivo and to estimate the *e*GI value [6]. The changes in the content of starch components during digestion are shown in Table 3. As the content of HBF and EHBF increased, the RDS content in fresh wet noodles decreased, while the SDS and RS contents increased. The levels of RDS and SDS serve as significant markers for assessing the *e*GI value of food. A study found that lower RDS and higher SDS levels contribute to blood glucose regulation and decrease the occurrence of certain cancers [26]. RS is generally fermented into short-chain fatty acids in the large intestine, rather than being digested in the small intestine [13]. The alterations in the contents of RDS, SDS, and RS suggested that fresh wet noodles with HBF and EHBF resisted gastrointestinal digestion. This phenomenon is attributed to the compositional differences between highland barley and wheat. HBF and EHBF, being rich in dietary fiber, hinder starch digestion by increasing the viscosity of intestinal contents and restricting the contact between digestive enzymes and substrates [19]. Additionally, phenols present in HBF and EHBF delay glucose release by inhibiting the activity of digestive enzymes such as α-amylase and α-glucosidase [14]. Fresh wet noodles containing EHBF exhibit higher RDS levels and lower SDS and RS levels compared to those containing HBF, suggesting that extrusion treatment enhances the digestibility of starch in highland barley. This aligns with the findings of Gao et al. [17], who reported that extrusion treatment disrupts the starch structure in buckwheat flour, facilitating enzyme access to the substrate.

The *e*GI value was determined by fitting the initial digestion curve to a first-order kinetic function. The change in the *e*GI value corresponded to the distribution pattern of each starch component during digestion (Table 3). Lower RDS levels and higher SDS and RS levels are associated with reduced *e*GI values. Fresh wet noodles with added HBF exhibited *e*GI values ranging from 72.46 to 78.16, whereas those with added EHBF showed *e*GI values between 79.45 and 82.54. A study found that foods with a *e*GI above 70 are categorized as high blood glucose foods, those with a *e*GI between 55 and 69 are medium blood glucose foods, and those with a *e*GI below 55 are categorized as low blood glucose foods [1]. The *e*GI of fresh wet noodles with HBF and EHBF exceeded 70, classifying them as high blood glucose foods. However, the *e*GI of fresh wet noodles enhanced with HBF and EHBF were significantly lower than those of the control group (84.80 ± 1.21) (*p* < 0.05), indicating that incorporating highland barley into fresh wet noodles is a suitable strategy for modulating digestibility and managing postprandial blood glucose levels.

### 3.7. Effects of HBF and EHBF Addition on the Texture Properties of Fresh Wet Noodles

Texturing is a highly effective method for assessing the eating quality of cooked fresh wet noodles [2]. The impact of HBF and EHBF on the texture properties of these noodles is detailed in Table 4. Relative to the control group, the incorporation of HBF and EHBF decreased the hardness and chewiness of the cooked noodles. The hardness and chewiness of cooked noodles are generally directly related to the gluten content [5]. By diluting the gluten present, the addition of HBF and EHBF led to softer and less chewy noodles. Specifically, the addition of EHBF significantly decreased the hardness compared to HBF (*p* < 0.05), probably due to the weakening of the protein by extrusion, which weakened the gluten network [31]. In contrast, changes in the adhesiveness of cooked fresh wet noodles were the opposite of those observed in hardness and chewiness. The adhesiveness increased with the inclusion of HBF and EHBF, except at the 10% EHBF level. Some studies have linked the adhesiveness of cooked noodles to cooking loss [17,25]. An increase in adhesiveness indicated that starch granules in HBF and EHBF are easily exudated to the surface of fresh wet noodles during cooking.

The effects of HBF and EHBF on the springiness and cohesiveness of cooked fresh wet noodles are also shown in Table 4. The springiness and cohesiveness of the cooked fresh wet noodles initially increased and then decreased with the addition of HBF and EHBF compared to that observed in the control group. The springiness and cohesiveness of cooked noodles are related to the stability of the noodle system [34]. Adding 10–20% HBF and EHBF can enhance the springiness and cohesiveness of the cooked fresh wet noodles by forming a stable starch–protein network structure. This finding aligned with the microstructure of fresh wet noodles (Figure 1). The resilience of cooked fresh wet noodles depends on the strength of the gluten network and the gelatinization properties of starch [2,27]. Adding HBF decreased the resilience of cooked fresh wet noodles compared to the control group, while adding EHBF increased it. This result is mainly due to the fact that HBF diluted gluten protein, leading to a weaker gluten network. In contrast, EHBF, through gelatinization during extrusion, enhanced the resilience of cooked fresh wet noodles.

### 3.8. Effects of Adding HBF and EHBF on the Sensory Evaluation of Fresh Wet Noodles

**Figure 4:**
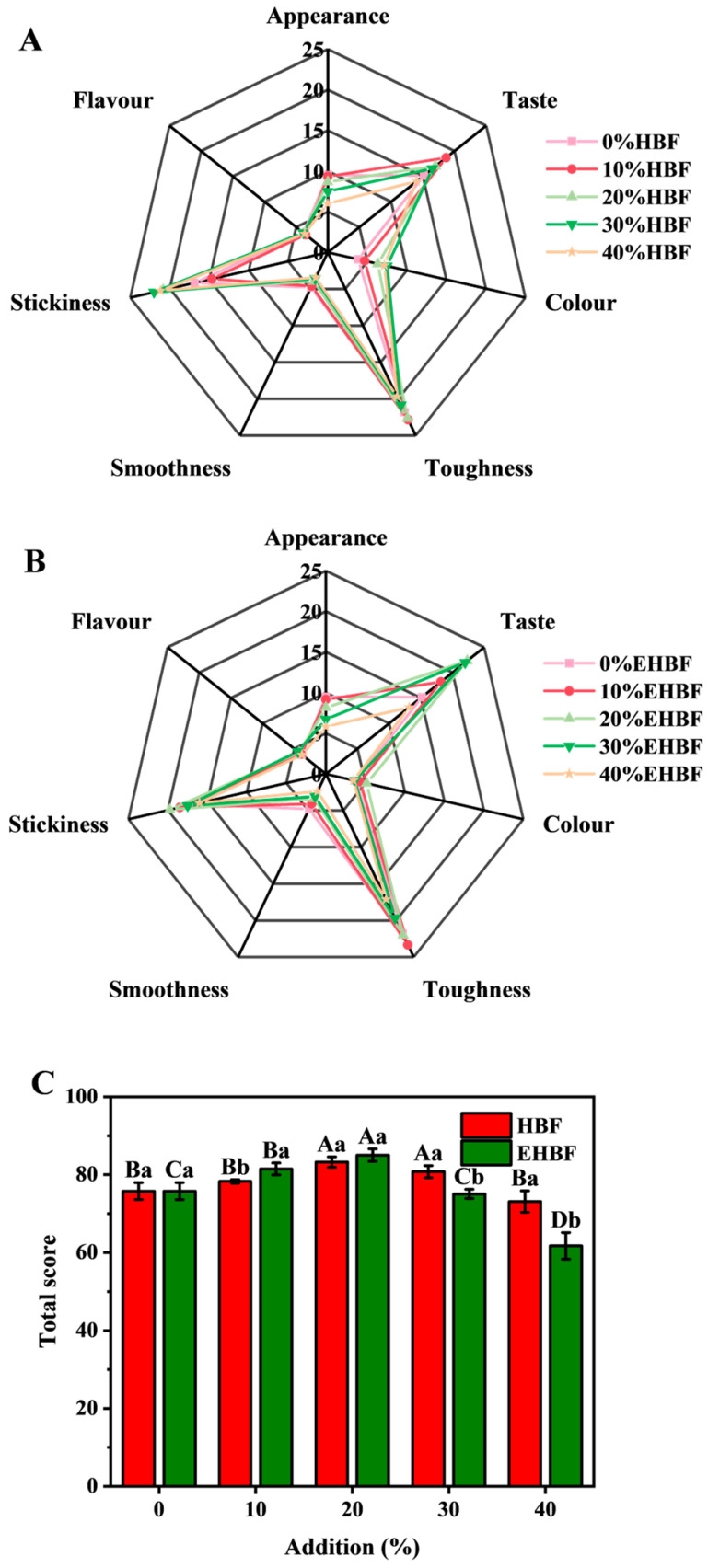
Effects of HBF (**A**) and EHBF (**B**) addition on the sensory evaluation of fresh wet noodles. Values show mean ± standard deviation (n = 3). Different samples with the same addition level are marked with different small letters (a, b) for the expression of the significant difference (*p* < 0.05). The same sample at a different addition level is marked with different capital letters (A, B, C, and D) for the expression of the significant difference (*p* < 0.05). HBF: highland barley flour; EHBF: extruded highland barley flour.

Sensory evaluation is another crucial method for assessing the eating quality of cooked fresh wet noodles [33]. The sensory evaluation results for appearance, taste, color, toughness, smoothness, stickiness, flavor, and overall score are shown in Figure 4. The taste, color, toughness, stickiness, flavor, and overall score of cooked fresh wet noodles first increased and then decreased with the addition of HBF and EHBF, compared to that recorded in the control group. These outcomes corresponded with changes in color, cooking quality, and texture observed in fresh wet noodles. Adding low amounts of HBF and EHBF helped maintain the color and stability of the dough, which increased the eating quality of cooked fresh wet noodles [20,34]. Additionally, adding HBF and EHBF led to a reduction in the appearance and smoothness of cooked fresh wet noodles. This effect may be attributed to the higher crude fiber content in HBF and EHBF, which decreased the visual appeal and smooth texture of cooked fresh wet noodles [35]. Cooked fresh wet noodles achieved the highest overall score when 20% EHBF was added, surpassing the score recorded after adding HBF. Extrusion processes can stimulate the Maillard reaction, producing flavor compounds that increase the eating quality of cooked fresh wet noodles [28]. Therefore, incorporating 20% EHBF in the production of fresh wet noodles may be beneficial.

## 4. Conclusions

The findings of this study indicated that adding EHBF enhanced the dough properties and quality characteristics of fresh wet noodles more effectively than adding HBF. The extrusion process gelatinized the starch in EHBF, improved the interaction within the starch chain in the amorphous region, and increased the wheat flour’s trough viscosity, final viscosity, and pasting temperature. Simultaneously, higher exposure of hydroxyl groups in EHBF increased the water absorption of the wheat flour dough, reducing both the dough development time and dough stability time. Adding a lower quantity of EHBF helped maintain the gluten network structure of fresh wet noodles and increased their cooking and sensory qualities. Adding 10–30% EHBF improved the springiness, cohesiveness, and resilience of the cooked fresh wet noodles. Although the effect of adding EHBF on reducing in vitro starch digestibility was not as good as that of HBF, it still effectively lowered the *e*GI of fresh wet noodles. Given the health benefits of highland barley, the outcomes of this study provide a strong scientific foundation for using EHBF in staple foods like noodles.

## Figures and Tables

**Table 1:** Effects of HBF and EHBF addition on the pasting properties of wheat flour.

Sample	Peak Viscosity (mPa·s)	Trough Viscosity (mPa·s)	Breakdown (mPa·s)	Final Viscosity (mPa·s)	Setback (mPa·s)	Pasting Temperature (°C)
Control	1653.67 ± 19.86 ^a^	1249.00 ± 20.88 ^a^	404.67 ± 40.43 ^c^	2369.67 ± 20.31 ^a^	1120.67 ± 33.50 ^a^	86.68 ± 0.78 ^c^
10% HBF	1434.33 ± 24.17 ^c^	947.67 ± 5.77 ^c^	486.67 ± 19.86 ^b^	1865.33 ± 22.72 ^c^	917.67 ± 17.50 ^b^	86.08 ± 0.45 ^c^
20% HBF	1280.00 ± 44.40 ^d^	759.67 ± 40.10 ^e^	520.33 ± 15.31 ^b^	1588.00 ± 60.65 ^e^	828.33 ± 29.02 ^c^	84.97 ± 0.03 ^c^
30% HBF	1110.67 ± 43.00 ^f^	594.67 ± 23.86 ^g^	516.00 ± 31.24 ^b^	1361.33 ± 46.09 ^fg^	766.67 ± 35.02 ^d^	84.45 ± 0.91 ^c^
40% HBF	1093.00 ± 27.62 ^f^	533.00 ± 29.05 ^h^	560.00 ± 4.58 ^a^	1302.00 ± 29.55 ^g^	769.00 ± 6.08 ^d^	84.20 ± 0.83 ^c^
10% EHBF	1524.67 ± 23.54 ^b^	1121.67 ± 33.56 ^b^	403.00 ± 22.52 ^c^	2079.33 ± 34.30 ^b^	957.67 ± 38.37 ^b^	87.47 ± 0.83 ^b^
20% EHBF	1320.67 ± 26.08 ^d^	934.00 ± 14.80 ^c^	386.67 ± 12.01 ^c^	1767.00 ± 34.77 ^d^	833.00 ± 19.97 ^c^	88.02 ± 0.49 ^ab^
30% EHBF	1201.00 ± 11.79 ^e^	828.67 ± 6.11 ^d^	372.33 ± 5.69 ^c^	1599.67 ± 13.65 ^e^	771.00 ± 7.55 ^d^	88.30 ± 0.10 ^a^
40% EHBF	1018.67 ± 46.48 ^g^	692.00 ± 34.66 ^f^	326.67 ± 11.85 ^d^	1378.00 ± 55.43 ^f^	686.00 ± 20.88 ^e^	88.25 ± 0.85 ^a^

Values show mean ± standard deviation (n = 3). Different letters in the same column are significantly different (*p* < 0.05). HBF: highland barley flour; EHBF: extruded highland barley flour.

**Table 2:** Effects of HBF and EHBF addition on the Mixolab parameters of wheat flour dough.

Sample	Water Absorption (%)	Protein Weakening (C2, Nm)	Starch Gelatinization (C3, Nm)	Starch Retrogradation (C5, Nm)	Dough Development (min)	Dough Stability (min)
Control	64.05 ± 1.34 ^de^	0.54 ± 0.01 ^a^	1.91 ± 0.01 ^a^	3.52 ± 0.01 ^a^	4.14 ± 0.16 ^a^	5.75 ± 0.33 ^a^
10% HBF	60.72 ± 1.49 ^e^	0.47 ± 0.00 ^b^	1.88 ± 0.02 ^a^	2.81 ± 0.24 ^b^	3.43 ± 0.11 ^ab^	4.09 ± 0.30 ^c^
20% HBF	60.92 ± 1.52 ^e^	0.46 ± 0.01 ^b^	1.88 ± 0.04 ^a^	2.56 ± 0.07 ^c^	3.35 ± 0.11 ^ab^	4.14 ± 0.44 ^c^
30% HBF	61.24 ± 1.41 ^e^	0.45 ± 0.02 ^b^	1.86 ± 0.06 ^a^	2.32 ± 0.06 ^d^	3.08 ± 1.20 ^bc^	5.19 ± 0.05 ^b^
40% HBF	61.71 ± 1.12 ^e^	0.45 ± 0.00 ^b^	1.84 ± 0.00 ^a^	2.21 ± 0.01 ^de^	1.54 ± 0.02 ^e^	5.73 ± 0.08 ^ab^
10% EHBF	67.35 ± 1.35 ^d^	0.40 ± 0.01 ^c^	1.65 ± 0.02 ^b^	2.72 ± 0.01 ^bc^	2.74 ± 0.27 ^cd^	3.19 ± 0.37 ^d^
20% EHBF	75.08 ± 1.56 ^c^	0.38 ± 0.00 ^cd^	1.49 ± 0.02 ^c^	2.05 ± 0.09 ^e^	2.14 ± 0.05 ^cd^	2.62 ± 0.16 ^e^
30% EHBF	82.35 ± 1.23 ^b^	0.37 ± 0.00 ^d^	1.28 ± 0.00 ^d^	1.63 ± 0.04 ^f^	2.02 ± 0.19 ^de^	2.39 ± 0.05 ^e^
40% EHBF	88.52 ± 1.38 ^a^	0.37 ± 0.02 ^d^	1.09 ± 0.04 ^e^	1.29 ± 0.03 ^g^	1.82 ± 0.07 ^de^	2.40 ± 0.03 ^e^

Values show mean ± standard deviation (n = 3). Different letters in the same column are significantly different (*p* < 0.05). HBF: highland barley flour; EHBF: extruded highland barley flour.

**Table 3:** Effects of HBF and EHBF addition on the starch digestibility of fresh wet noodles.

Sample	RDS (%)	SDS (%)	RS (%)	HI (%)	*e*GI
Control	61.74 ± 1.23 ^a^	28.92 ± 0.82 ^g^	9.33 ± 0.41 ^e^	82.14 ± 2.55 ^a^	84.80 ± 1.21 ^a^
10% HBF	55.09 ± 1.15 ^de^	32.41 ± 0.61 ^d^	12.20 ± 0.41 ^b^	70.03 ± 1.82 ^c^	78.16 ± 1.48 ^d^
20% HBF	53.61 ± 0.64 ^e^	33.79 ± 0.31 ^c^	12.59 ± 0.31 ^b^	70.34 ± 1.99 ^c^	78.33 ± 1.46 ^d^
30% HBF	50.63 ± 1.21 ^f^	36.77 ± 0.82 ^b^	12.59 ± 0.46 ^b^	64.69 ± 2.25 ^d^	75.22 ± 1.74 ^e^
40% HBF	48.13 ± 1.39 ^g^	38.34 ± 0.21 ^a^	13.52 ± 0.23 ^a^	59.65 ±1.45 ^e^	72.46 ± 1.43 ^f^
10% EHBF	59.38 ± 0.53 ^b^	30.22 ± 0.37 ^f^	10.40 ± 0.32 ^d^	78.03 ± 1.42 ^ab^	82.54 ± 1.65 ^b^
20% EHBF	57.87 ± 0.95 ^bc^	30.66 ± 0.93 ^ef^	11.46 ± 0.45 ^c^	76.19 ± 2.23 ^b^	81.54 ± 1.42 ^b^
30% EHBF	55.89 ± 0.86 ^d^	31.66 ± 0.51 ^de^	12.45 ± 0.26 ^b^	74.53 ± 1.93 ^b^	80.63 ± 1.15 ^bc^
40% EHBF	56.17 ± 0.97 ^cd^	31.46 ± 0.79 ^de^	12.37 ± 0.15 ^b^	72.38 ± 2.75 ^c^	79.45 ± 1.39 ^cd^

Values show mean ± standard deviation (n = 3). Different letters in the same column are significantly different (*p* < 0.05). HBF: highland barley flour; EHBF: extruded highland barley flour; RDS: rapidly digestible starch; SDS: slowly digestible starch; RS: resistant starch; HI: hydrolysis index; *e*GI: estimated glycemic index.

**Table 4:** Effects of HBF and EHBF addition on the texture characteristics of fresh wet noodles.

Sample	Hardness (N)	Adhesiveness (N·s)	Springiness	Cohesiveness	Chewiness (N)	Resilience
Control	2187.94 ± 55.19 ^a^	−107.35 ± 30.82 ^bc^	0.84 ± 0.04 ^b^	0.58 ± 0.02 ^cd^	1069.32 ± 96.64 ^a^	0.24 ± 0.01 ^c^
10% HBF	1772.18 ± 20.89 ^b^	−69.77 ± 33.32 ^ab^	0.92 ± 0.02 ^a^	0.60 ± 0.02 ^bc^	973.35 ± 37.32 ^b^	0.23 ± 0.02 ^cd^
20% HBF	1621.81 ± 10.44 ^c^	−59.41 ± 12.54 ^ab^	0.90 ± 0.03 ^ab^	0.60 ± 0.02 ^bc^	880.95 ± 68.61 ^b^	0.21 ± 0.01 ^de^
30% HBF	1565.83 ± 36.29 ^c^	−48.49 ± 27.84 ^a^	0.75 ± 0.05 ^c^	0.50 ± 0.02 ^e^	580.62 ± 63.50 ^d^	0.20 ± 0.02 ^ef^
40% HBF	1465.00 ± 10.80 ^d^	−55.00 ± 22.35 ^a^	0.75 ± 0.02 ^c^	0.50 ± 0.02 ^e^	545.11 ± 30.84 ^d^	0.18 ± 0.01 ^f^
10% EHBF	1620.22 ± 52.17 ^c^	−145.02 ± 59.51 ^c^	0.90 ± 0.02 ^ab^	0.63 ± 0.01 ^a^	928.58 ± 54.05 ^b^	0.28 ± 0.01 ^ab^
20% EHBF	1401.81 ± 6.32 ^e^	−92.13 ± 13.16 ^ab^	0.89 ± 0.02 ^ab^	0.62 ± 0.01 ^ab^	775.99 ± 14.83 ^c^	0.27 ± 0.01 ^b^
30% EHBF	1365.96 ± 35.05 ^e^	−79.14 ± 17.19 ^ab^	0.88 ± 0.05 ^ab^	0.61 ± 0.01 ^ab^	727.03 ± 37.03 ^c^	0.31 ± 0.01 ^a^
40% EHBF	1247.03 ± 43.79 ^f^	−91.68 ± 0.67 ^ab^	0.85 ± 0.05 ^b^	0.57 ± 0.01 ^d^	605.92 ± 16.34 ^d^	0.24 ± 0.02 ^c^

Values show mean ± standard deviation (n = 3). Different letters in the same column are significantly different (*p* < 0.05). HBF: highland barley flour; EHBF: extruded highland barley flour.

## Data Availability

The original contributions presented in the study are included in the article, further inquiries can be directed to the corresponding author.
